# Inhibition of DNA-dependent protein kinase catalytic subunit boosts rAAV transduction of polarized human airway epithelium

**DOI:** 10.1016/j.omtm.2023.101115

**Published:** 2023-09-21

**Authors:** Kang Ning, Xiujuan Zhang, Zehua Feng, Siyuan Hao, Cagla Aksu Kuz, Fang Cheng, Soo Yuen Park, Shane McFarlin, John F. Engelhardt, Ziying Yan, Jianming Qiu

**Affiliations:** 1 Department of Microbiology, Molecular Genetics and Immunology, University of Kansas Medical Center, Kansas City, KS 66160, USA; 2 Department of Anatomy and Cell Biology, University of Iowa, Iowa City, IA 52242, USA

**Keywords:** rAAV, transduction, DNA damage response, DNA-PK_cs_, human airway epithelium

## Abstract

Adeno-associated virus 2.5T (AAV2.5T) was selected from the directed evolution of AAV capsid library in human airway epithelia. This study found that recombinant AAV2.5T (rAAV2.5T) transduction of well-differentiated primary human airway epithelia induced a DNA damage response (DDR) characterized by the phosphorylation of replication protein A32 (RPA32), histone variant H2AX (H2A histone family member X), and all three phosphatidylinositol 3-kinase-related kinases: ataxia telangiectasia mutated kinase, ataxia telangiectasia and Rad3-related kinase (ATR), and DNA-dependent protein kinase catalytic subunit (DNA-PK_cs_). While suppressing the expression of ATR by a specific pharmacological inhibitor or targeted gene silencing inhibited rAAV2.5T transduction, DNA-PK_cs_ inhibition or targeted gene silencing significantly increased rAAV2.5T transgene expression. Notably, DNA-PK_cs_ inhibitors worked as a “booster” to further increase rAAV2.5T transgene expression after treatment with doxorubicin and did not compromise epithelial integrity. Thus, our study provides evidence that DDR is associated with rAAV transduction in well-differentiated human airway epithelia, and DNA-PK_cs_ inhibition has the potential to boost rAAV transduction. These findings highlight that the application of DDR inhibition-associated pharmacological interventions has the potential to increase rAAV transduction and thus to reduce the required vector dose.

## Graphical abstract

**Figure:**
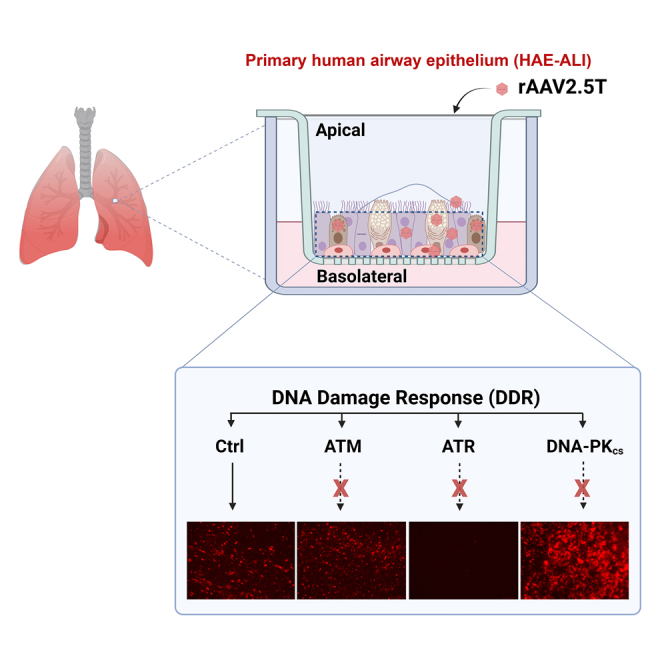

## Teaser

Dr. Qiu and colleagues report that AAV2.5T transduction of well-differentiated primary human airway epithelia induces a DNA damage response, including activation of ATM, ATR, and DNA-PK_cs_. ATR inhibition inhibits rAAV2.5 transduction, whereas ATM inhibition has no effects. Strikingly, DNA-PK_cs_ inhibition alone or in a combination with doxorubicin boosts rAAV2.5T transduction.

## Introduction

Recombinant adeno-associated virus (rAAV) has been used as a gene transfer vector for human gene therapy of several genetic diseases.^1^^,^^2^ To date, three rAAV-based gene therapy products have been approved by the FDA, i.e., Hemgenix (hemophilia B),^3^ Luxturna (retinal dystrophy),^4^ and Zolgensma (spinal muscular atrophy).^5^ rAAV vectors are able to deliver transgene to both dividing and non-dividing cells.^6^^,^^7^ Due to the persistence of their episomal genomes, long-term expressions are achievable in differentiated cells/tissues, such as the skeletal and cardiac muscle cells,^8^^,^^9^ neurons,^10^ retina,^11^ human airway epithelia (HAE),^12^ and hepatocytes.^13^ Some rAAV serotypes have demonstrated their ability to transduce stem cells, e.g., CD34^+^ human hematopoietic stem cells (HSCs)^14^ and induced pluripotent stem cells, indicating their potential application for therapeutic genome editing in stem cells.

Parvovirus infection activates DNA damage response (DDR), which is essential for parvovirus replication in host cells.^15^^,^^16^ In differentiated HAE, human bocavirus 1 (HBoV1) infection initiates a DDR with the activation of all three phosphatidylinositol 3-kinase-related kinases (PIKKs): ataxia telangiectasia and Rad3-related kinase (ATR), ataxia telangiectasia mutated kinase (ATM), and DNA-dependent protein kinase catalytic subunit (DNA-PK_cs_), and the DNA repair DNA polymerases involved in HBoV1 viral DNA replication.^17^ DNA-PK plays an important role in viral DNA replication, which is due in part to the interaction of Ku70 with the small viral nonstructural protein NP.^18^ The role of activation of the three PIKKs has also been confirmed in wild-type AAV (wtAAV) monoinfection of human embryonic kidney 293T (HEK293T) cells, with DNA repair polymerases also contributing to wtAAV DNA replication.^19^ DDR induced by wtAAV monoinfection is cell-type specific. While UV (ultraviolet)-inactivated AAV genomes mimic a stalled replication fork and promote a DDR that is restricted to ATR and Chk1,^20^ during monoinfection of wtAAV2, viral DNA replication, but not single-stranded DNA (ssDNA) genome accumulation and Rep expression, induces a robust DDR in HEK293T cells.^19^ In dividing cells, the DDR induced by the uptake of UV-inactivated AAV activates p53 and arrests cells in the G2/M phase of the cell cycle.^21^^,^^22^

The extent to which rAAV transduction induces a DDR is still a matter of debate and ongoing research. rAAV transduction in U2OS cells is largely deficient in activating a DDR,^23^ whereas in CD34^+^ HSCs it triggers a p53-mediated DDR.^24^^,^^25^ It has been reported that rAAV transduction is accompanied with some toxicity, which might be due to DNA damage or induced genotoxic stress.^26^^,^^27^^,^^28^ After cellular uptake and nuclear transport of the rAAV genome, the 3′ OH group of the inverted terminal repeats (ITRs) functions as a primer for the second-strand synthesis by host DNA replication and/or repair DNA polymerases, which is independent of cell-cycle arrest or cell division.^27^^,^^29^ The duplex rAAV genome is capable of long-term persistence as a linear double-stranded DNA (dsDNA) genome or a circular episome in the form of monomers or concatemers.^6^^,^^7^^,^^9^^,^^30^ Both circularization and concatemerization of rAAV genomes have been reported to occur through the major host DNA repair pathways, non-homologous end joining (NHEJ), and homologous recombination (HR). While NHEJ relies on the activation of DNA-PK, HR is dependent on ATM activation.^29^^,^^31^^,^^32^ Previous studies found that degradation of Mre11, Rad50, and Nbs1 (MRN), the cellular DNA damage-sensing complex, enhances gene expression from both single- and double-stranded rAAV vectors. However, this inhibition of rAAV-mediated gene expression by MRN does not require downstream DDR factors, including signaling kinases ATM and ATR.^33^ In addition, it was reported that the transgene expression from an oversized rAAV (∼6.2 kb) transduction is mediated by the recombination of “fragment” AAV genomes, which is independent of the DNA-PK_cs_ repair pathway, but reliant on Rad51C, a DNA strand-transfer protein involved in annealing during HR.^27^ Whether rAAV transduction of polarized HAE that are comprised of well-differentiated epithelial cells and mitotically quiescent basal cells induces a DDR has yet to be examined.

The natural tropism of AAV can be cell-type specific or broad, depending on serotypes.^34^ Advances in AAV capsid-directed evolution and rational design have created many clinically desirable capsid variants that exhibit higher transduction efficiency to target disease-related tissues or cells for gene therapy.^14^^,^^35^^,^^36^^,^^37^ AAV2.5T is a capsid variant that was evolved from HAE cultured at an air-liquid interface (HAE-ALI).^38^ The AAV2.5T capsid is a chimera of the VP1 unique region of AAV2 and the VP2/3 regions of AAV5 with a single A581T mutation. rAAV2.5T transduces polarized HAE from the apical membrane with over 10-fold higher efficiency than rAAV2 and rAAV5. Impaired intracellular trafficking is a significant barrier to achieve productive rAAV transduction, particularly following infection of polarized HAE cultures from the apical surface. To overcome this obstacle, pharmacological interventions have been developed to improve various post-entry steps, including endosomal processing, nuclear import, uncoating of the virion, and dsDNA conversion. For example, tripeptidyl aldehyde proteasome inhibitors have been shown to effectively increase transduction of rAAV,^39^^,^^40^ while doxorubicin (Dox) has been shown to significantly enhance the transduction of different rAAV serotype vectors in polarized airway epithelia by over 100-fold through improving the efficiency of virion nuclear transport.^41^

In this study, we investigated the DDR activation following rAAV2.5T transduction of well-differentiated/polarized primary HAE-ALI. We found that induction of DDR occurred following rAAV2.5T infection, which was characterized by the phosphorylation of replication protein A32 (RPA32) and histone variant H2AX (H2A histone family member X), as well as all three PIKKs: ATM, ATR, and DNA-PK_cs_. Furthermore, we found that DNA-PK_cs_ inhibition or targeted gene knockdown increased rAAV2.5T transgene expression significantly in HAE-ALI and that specific inhibitors of DNA-PK_cs_ can work synergistically with Dox to boost rAAV transduction.

## Results

### rAAV transduction induces a DDR in well-differentiated primary HAE, but not in dividing HEK293, HeLa, and human airway epithelial (CuFi-8) cells

The induction of DDR by wtAAV is cell type dependent, whether rAAV transduction also activates a DDR remains unclear. While we found that wtAAV replication in HEK293T cells induces a robust DDR,^19^ studies on rAAV2.5T transduction in HEK293T HeLa as well as dividing cell lines CuFi-8 cells showed that the transductions of rAAV2.5TmCherry-firefly luciferase (rAAV2.5TmCfLuc) in these cell types induced negligible activation of DDR hallmarks, p-RPA32 and ꝩH2AX (Figure S1). Notably, rAAV2.5TmCfLuc efficiently transduces HEK293T cells.

**Figure 1:**
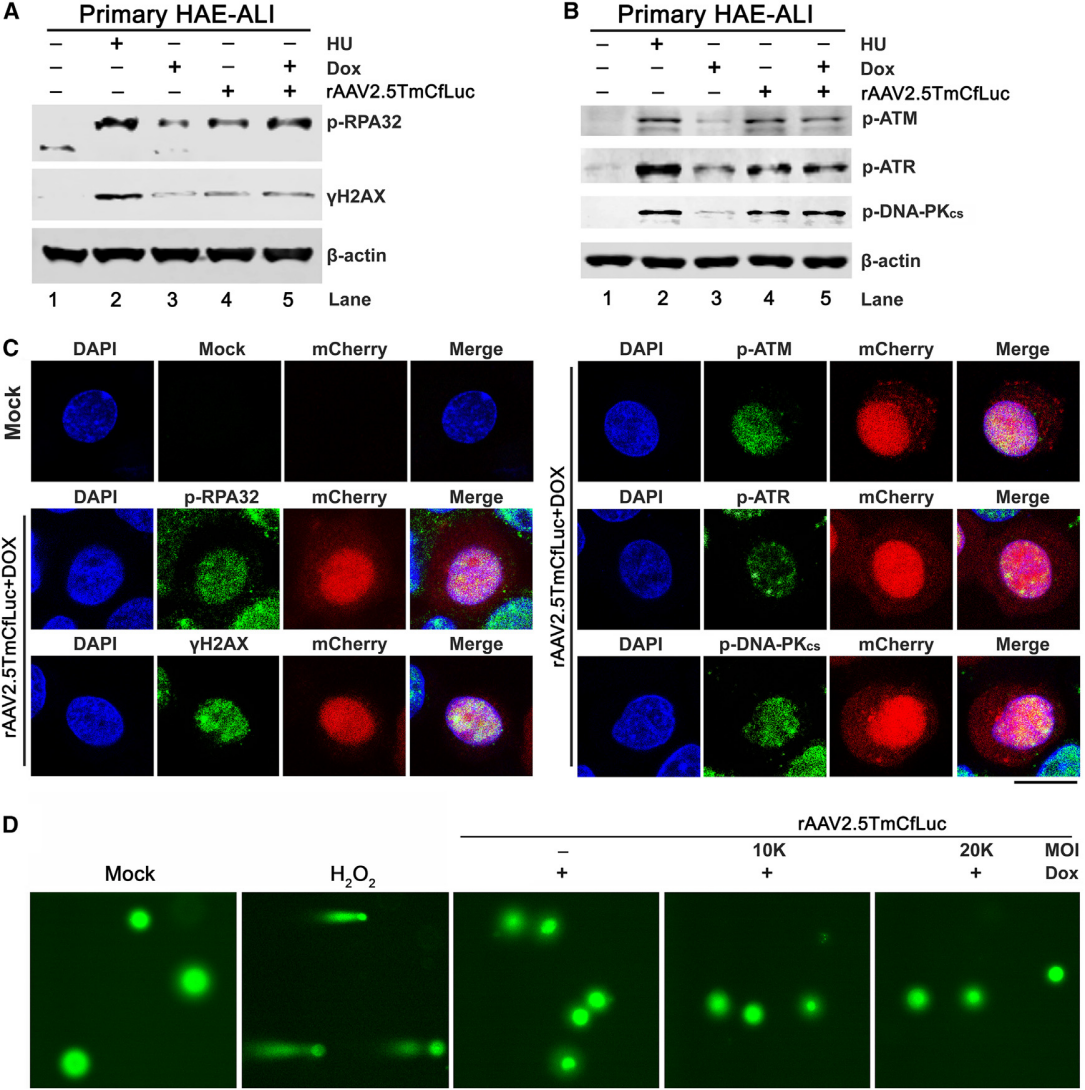
rAAV transduction of primary HAE-ALI induces DDR signals and activates ATM, ATR, and DNA-PK_cs_ Well-differentiated primary HAE-ALI cultures (derived from donor B13-40) were transduced with rAAV2.5TmCfLuc at an MOI of 10k DNase-resistant particles per cell. At 3 days post-transduction (dpt), the cells were collected for western blotting (A and B) and immunofluorescence assays (C) with antibodies against the indicated proteins. Anti-phospho(p)RPA32 (Thr21), anti-ꝩH2AX (Ser139), anti-p-ATM(S1981), anti-p-ATR(T1989), and anti-p-DNA-PK_cs_(S2056) were used for the detection of the phosphorylated forms of RPA32, H2AX, ATM, ATR, and DNA-PK_cs_ proteins, respectively. Hydroxyurea (HU)-treated cells served as a DDR-positive control and β-actin served as loading control. Scale bar, 25 μm. (D) Comet assay. At 3 dpt, the cells were collected for the comet assay; 100 μM hydrogen peroxide (H_2_O_2_)-treated cells were used as positive control for the comet assay. Data shown are representative of three independent experiments.

Polarized HAE-ALI cultures are differentiated from primary human bronchial epithelial cells and provide an *in vitro* model of HAE composed of multiple epithelial cell types.^42^ HAE-ALI mimics the structure and function of the *in vivo* proximal airway epithelium. While rAAV2.5T efficiently transduces HAE-ALI from the apical membrane, effective transduction requires the pharmacological intervention of Dox to overcome the post-entry block of viral nuclear entry.^41^ To investigate whether rAAV transduction in well-differentiated airway epithelia induces a DDR, we apically infected primary HAE-ALI cultures with rAAV2.5TmCfLuc at a multiplicity of infection (MOI) of 10,000 (10k) DNase-resistant particles per cell with or without 2.5 μM Dox applied to the medium in a basolateral chamber during the infection period. At 3 days post-transduction (3 dpt), western blotting detected two DDR hallmarks, phosphorylated RPA32 at threonine 21 (p-RPA32) and phosphorylated H2AX at serine 139 (ꝩH2AX) in the transduced cultures without Dox treatment (Figure 1A, lane 4). This result indicated that rAAV2.5TmCfLuc transduction of primary HAE-ALI induces a DDR. Dox-treated rAAV2.5TmCfLuc transduction also induced similar levels of expression of both p-RPA32 and ꝩH2AX (Figure 1A, lane 5 vs. 4). The application of Dox alone also induced p-RPA32 and ꝩH2AX expression, but to a relatively weaker extent, compared with the induction by rAAV2.5TmCfLuc (Figure 1A, lane 3).

Next, we confirmed that rAAV2.5TmCfLuc transduction of primary HAE-ALI without Dox treatment activated all three PIKKs including ATM, ATR, and DNA-PK_cs_ at a level similar to the group with rAAV and Dox treatment (Figure 1B, lane 4 vs. 5). Moreover, immunofluorescence staining further confirmed the activation of two DDR hallmarks and all three PIKKs in rAAV2.5TmCfLuc-transduced cells (mCherry positive) isolated from the infected primary HAE-ALI (Figure 1C). To investigate if the activation of DDR was the result of cellular DNA damage, we used comet assays to probe for damaged cellular DNA in rAAV2.5TmCfLuc-transduced HAE-ALI. The results showed that the transduction with Dox treatment only activated the DDR signals but did not obviously damage cellular DNA, even with increased vector load at an MOI of 20k with 2.5 μM Dox (Figure 1D). As a control, when H_2_O_2_ was added at 100 μM,^43^ the cells appeared comet positive (Figure 1D, H_2_O_2_/+), indicating the presence of broken host DNA. Thus, we concluded that rAAV2.5TmCfLuc transduction alone or in the presence of Dox activates a DDR in HAE-ALI without causing significant damage to the host chromosomal DNA, as detected by the comet assay.

Together with the fact that Dox treatment alone induced a relatively weaker activation of the three PIKKs, compared with rAAV2.5T transduction in the presence or absence of Dox (Figure 1B, lanes 3 vs. 4 and 5), our results demonstrated that the transduction of rAAV2.5T induced a DDR in well-differentiated HAE, but not in dividing HEK293T, HeLa, and CuFi-8 cells.

### Inhibition of DNA-PK_cs_ significantly enhances rAAV transduction in well-differentiated HAE: Conversely, ATR inhibition diminishes the transduction, while ATM inhibition has no effects

**Figure 2:**
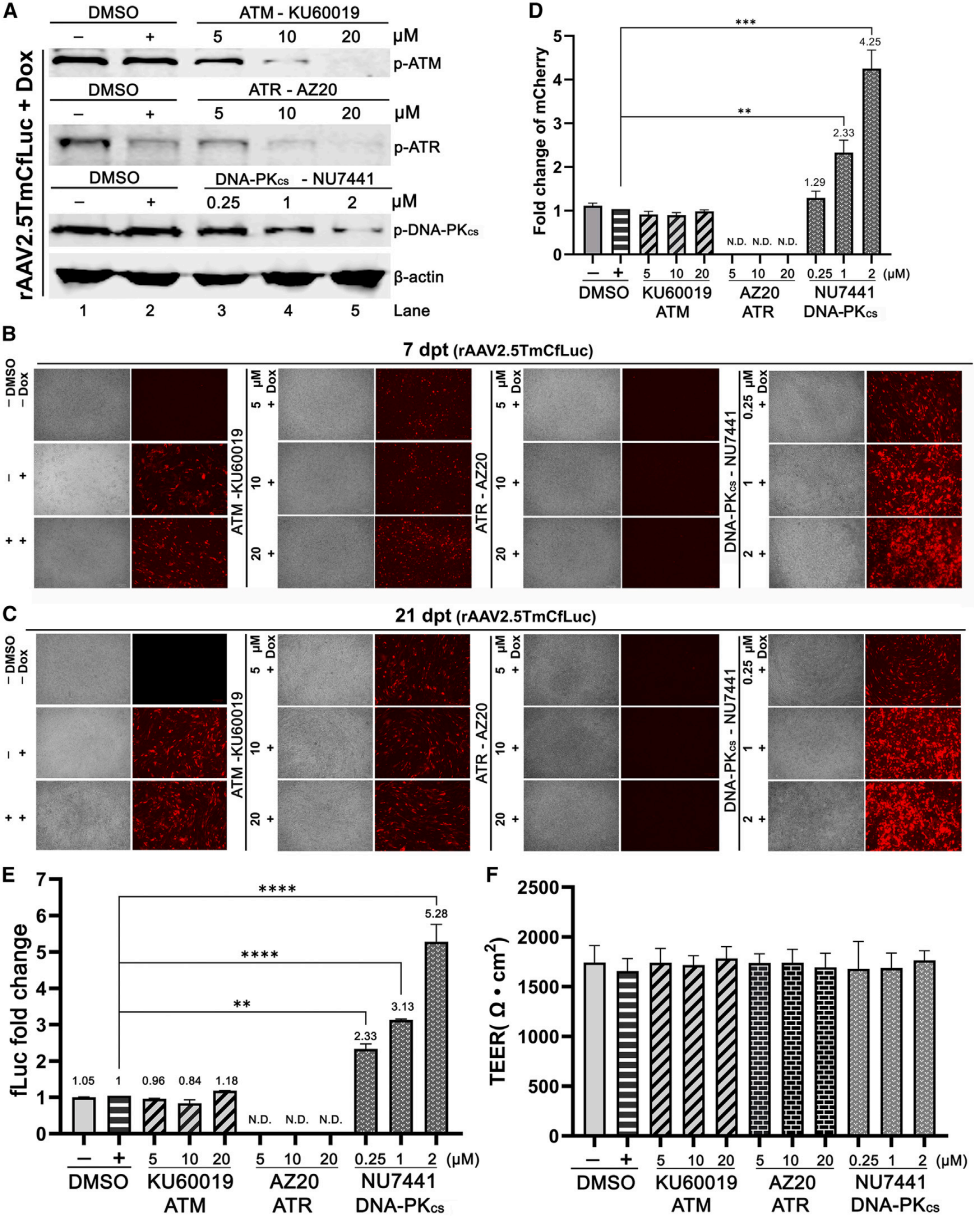
Treatment of ATM, ATR, and DNA-PK_cs_ pharmacological inhibitors alters rAAV transduction efficiency in primary HAE-ALI At 4 h prior to rAAV2.5TmCfLuc transduction (MOI = 10k, with 2.5 μM Dox), primary HAE-ALI cultures (B13-40) were treated with pharmacological inhibitors targeting ATM (KU60019), ATR (AZ20), and DNA-PK_cs_ (NU7441), respectively, using the indicated concentrations. Dimethylsulfoxide (DMSO) served as vehicle control. (A) Western blotting. At 3 dpt, the cells were collected for western blotting using specific antibodies targeting phosphorylated (p)-ATM, p-ATR, and p-DNA-PK_cs_, respectively. β-Actin served as a loading control. (B–D) mCherry expression. The mCherry expression was measured at 7 dpt (B) and 21 dpt (C), respectively, by the ZOE Fluorescent Cell Imager (Bio-Rad). The relative fold changes of mCherry expression in the treated ALI cultures to the DMSO control were measured by ImageJ (D). Data with means and standard deviations were obtained from three independent experiments. (E) Quantification of luciferase activity. At 21 dpt, the treated cells were lysed and the relative fold changes of luciferase activity to DMSO-treated cells were evaluated. The luciferase activity value of the DMSO-treated cells was arbitrarily set to 1. Relative folds to the DMSO control group are shown with means and standard deviations obtained from three independent experiments. (F) Measurement of the transepithelial electrical resistance (TEER). The indicated TEER values were measured by using an epithelial Volt-Ohm Meter (Millipore) on 21 dpt. Data shown are the averages and standard deviations of the TEER values obtained from three independent experiments. p values were analyzed by using Student’s t test (∗∗p < 0.01, ∗∗∗∗p < 0.0001). N.D., not detectable.

We next explored the role of PIKK activation in rAAV transduction of well-differentiated primary HAE-ALI derived from the primary bronchial airway cells of donor B13-40. The ALI cultures were first treated with their respective kinase-specific pharmacological inhibitor for 4 h, followed by rAAV2.5TmCfLuc transduction at an MOI of 10k in the presence of 2.5 μM Dox in the basolateral chamber during the infection period. At 3 dpt, western blot confirmed the application of each inhibitor, KU60019 (specific to ATM), AZ20 (specific to ATR), or NU7441 (specific to DNA-PK_cs_), efficiently and specifically inhibited the phosphorylation of the corresponding targeted kinase (Figure 2A). Reporter mCherry expression was continually monitored during the experiment course, and the representative images captured at 7 and 21 dpt (Figures 2B and 2C). While the inhibition of ATM kinase showed negligible impact on rAAV transgene expression, notably different effects from the inhibitions of ATR and DNA-PK_cs_ were observed. The inhibition of ATR kinase almost completely blocked the rAAV transgene expression, while the treatment of a DNA-PK_cs_ inhibitor, NU7441, significantly increased the mCherry expression in a dose-dependent manner (Figure 2D). More accurate quantitation for the fold changes in transgene expression was obtained from the measurement of firefly luciferase (fLuc) activity in the cell lysates when the experiment was terminated at 21 dpt (Figure 2E).

To exclude the variables from vector prep and donor dependence of the primary cultures, the NU7441 enhancement of rAAV2.5TmCfLuc transduction was confirmed with the use of another transgene expressing rAAV2.5T and primary HAE-ALI prepared from primary bronchial cells of another donor (B40-22). In this test, we used rAAV2.5TmCherrygLuc, which expresses the secreting *Gaussia* luciferase (gLuc), enabling us to continually monitor both mCherry and gLuc expression over the course of the experiment. We observed that the transgene expressions from this dual reporter vector were increased by treatment of NU7441 at 0.25 to 2 μM in a dose-dependent manner at 7, 14, and 21 dpt, echoing the results from the HAE-ALI derived from donor B13-40 described above (Figures S2A and S2B). Notably, during the experiments, the transepithelial electrical resistance (TEER) values of the inhibitor-treated primary HAE-ALI remained >1,500 Ω cm^2^, indicating an intact epithelial barrier function and a low cytotoxicity caused by these compounds at the indicated concentrations (Figures 2F and S2C).

### Silencing of *DNA-PK*_*cs*_ substantially enhances rAAV transduction in well-differentiated airway epithelia, but not *ATM* or *ATR* silencing

**Figure 3:**
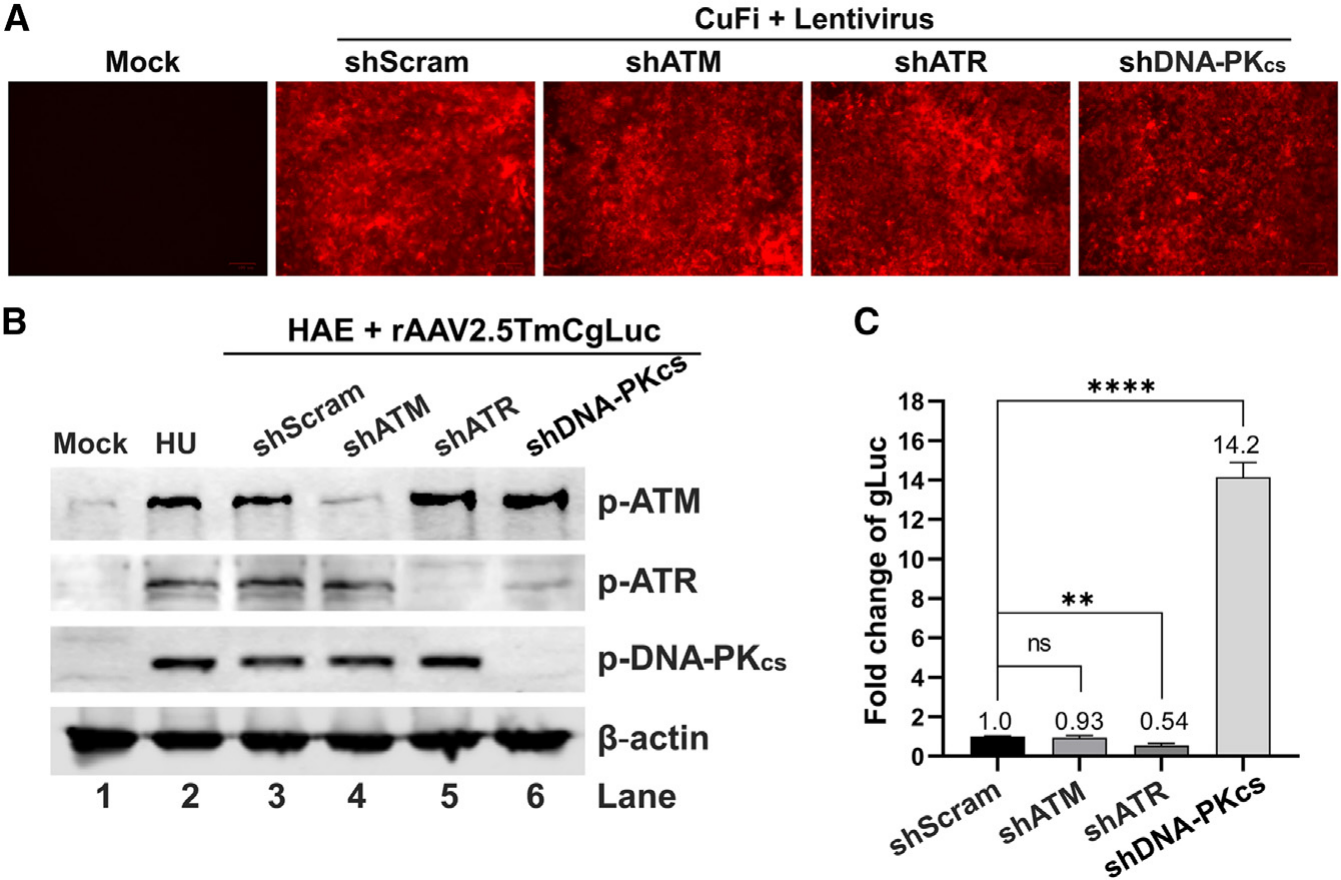
Knockdown of ATM, ATR, and DNA-PK_cs_ affects rAAV transduction Dividing CuFi-8 cells were transduced with lentivirus expressing DDR kinase-targeted shRNA or scramble shRNA as indicated prior to differentiation. The transduced cells were then applied to transwells for differentiation at an ALI for 28 days. Well-differentiated HAE-ALI with a TEER > 1,500 Ω cm^2^ were selected for experiments. (A) Fluorescence imaging. Before rAAV transduction, mock and shRNA/mCherry-expressing HAE-ALI cultures were visualized for mCherry expression under a Bio-Rad ZOE Fluorescent Cell Imager. (B) Western blotting. At 5 dpt, the transduced HAE-ALI cultures were collected and lysed for western blotting using anti-p-ATM(S1981), anti-p-ATR(T1989), and anti-p-DNA-PK_cs_(S2056) antibodies, respectively. β-Actin was used as a loading control. (C) Quantification of luciferase activity. At 21 dpt, the luciferase activity of each group was measured and normalized to shScramble (shScram) transduced group. p values were calculated by using Student’s t test (∗∗p < 0.01, ∗∗∗∗p < 0.0001; n.s., no statistically significant difference). The data shown are means and standard deviations obtained from three independent experiments.

We next used an shRNA-mediated gene-silencing strategy to knock down the expression of ATM, ATR, and DNA-PK_cs_, respectively, and examined the specific gene silencing on rAAV transduction in well-differentiated HAE-ALI cultures. To this end, a set of lentiviral vectors, which co-expressed each shRNA with an mCherry reporter and has previously showed success in silencing these genes,^17^^,^^19^ was used to infect CuFi-8 cells, an immortalized human airway epithelial cell line maintaining the potential to differentiate into polarized HAE-ALI.^44^ Monolayer cultures of proliferating CuFi-8 cells were transduced with an individual lentiviral vector for targeted gene silencing prior to polarization at an ALI for cell differentiation. As expected, these lentiviruses showed a high transduction efficiency, indicated by the mCherry reporter expression in differentiated ALI cultures (Figure 3A). These transduced HAE-ALI cultures were then transduced with rAAV2.5TmCgLuc. At 5 dpt, we examined the targeted gene silencing and the activation of the three PIKKs from the rAAV2.5T transduction by western blotting, confirming the activation of PIKKs associated with the efficient and specific knockdown of the expression of ATM, ATR, or DNA-PK_cs_ by the corresponding lentiviral vector compared with the scramble shRNA control (shScram), where all three PIKKs were activated (Figure 3B). At 21 dpt, the quantification of luciferase reporter activity showed that rAAV2.5T-mediated transgene expression in DNA-PK_cs_-knockdown epithelia was significantly increased by ∼14-fold; however, ATR-knockdown cells had a decrease in luciferase activity by ∼50%, and the ATM-knockdown cells had no significant changes (Figure 3C).

Collectively, these results confirmed that the expression of the rAAV transgene is related to PIKK activation; while ATR knockdown decreases rAAV transduction, DNA-PK_cs_ knockdown boosts rAAV transduction (by 14-fold), and ATM knockdown has no effect.

### DNA-PK_cs_ inhibition by NU7441 enhances rAAV transduction in well-differentiated HAE

**Figure 4:**
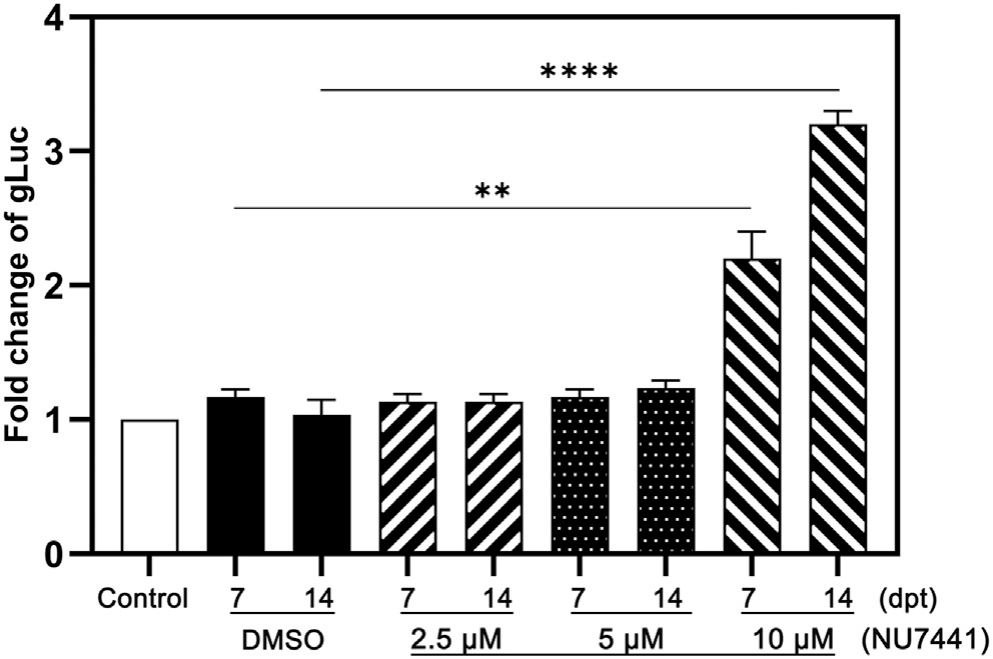
Monotreatment of NU7441 increases rAAV transgene expression Well-differentiated primary HAE-ALI cultures (B13-40) were transduced by rAAV2.5TmCgLuc (MOI = 10k) together with NU7441 at the indicated concentrations. At 7 and 14 dpt, the transgene expressions were measured by detection of the gLuc activity. The gLuc expression in only rAAV-infected primary HAE-ALI cultures was set to 1 (control). Results shown are means and standard deviations (n = 3) from three independent experiments. p values were calculated by using Student’s t test (∗∗p < 0.01, ∗∗∗∗p < 0.0001).

We next investigated whether NU7441 treatment alone enhances rAAV transduction in the absence of Dox. To this end, primary HAE-ALI cultures were treated with NU7441 only at 4 h prior to rAAV2.5TmCgLuc transduction (MOI = 10k) at 2.5, 5, and 10 μM, respectively. After overnight treatment, media were refreshed and gLuc activity was measured at 7 and 14 dpt. The results showed that the mono-treatment of NU7441 increased gLuc expression by ∼3-fold at 10 μM at 14 dpi compared with mock-treated group with the vehicle dimethyl sulfoxide (DMSO), which further confirmed the effectiveness of NU7441 in enhancing rAAV2.5TmCgLuc transduction in primary HEA-ALI (Figure 4).

**Figure 5:**
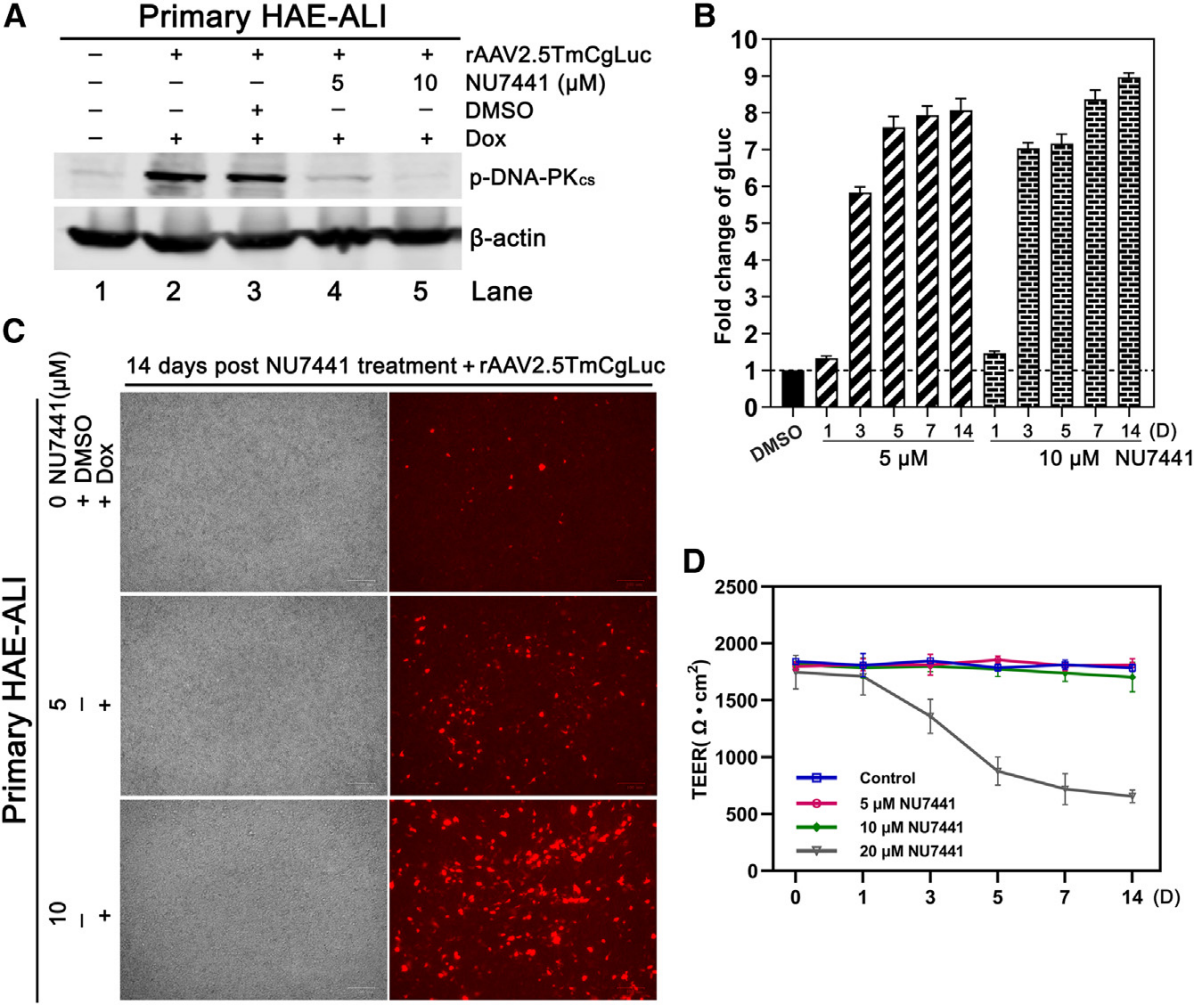
NU7441 functions as a “booster” with doxorubicin to further increase rAAV transgene expression Primary HAE-ALI cultures (B13-40) were transduced by rAAV2.5TmCgLuc at an MOI of 2.5k with the treatment of doxorubicin at 2.5 μM. At 7 dpt, the primary HAE-ALI cultures were further treated with NU7441 at the indicated concentrations. The media were refreshed after overnight or treatment of rAAV2.5T with Dox and NU7441. (A) Western blotting. The cells of the primary HAE-ALI cultures were collected and lysed for western blotting at 16 h post-treatment with NU7441. (B and C) Transgene expression quantification. The expressions of transgene (gLuc and mCherry) were measured as indicated. The fold changes of gLuc activity in NU7441-treated groups were normalized to the DMSO control group. (D) Measurement of TEER. The TEER of NU7441-treated primary HAE-ALI was measured at 0, 1, 3, 5, 7, and 14 days after NU7441 treatment, respectively. Results shown are means and standard deviations (n = 3) from three independent experiments.

To further investigate the impact of DNA-PK_cs_ inhibition on rAAV2.5TmCgLuc transduction in combination with Dox, we conducted additional experiments that applied DNA-PKcs inhibitor NU7441 at 7 dpt. To this end, primary HAE-ALI cultures were first transduced with rAAV2.5TmCgLuc at an MOI of 2.5k and treated with Dox at 2.5 μM during the infection period. At 7 dpt, which was defined as day 0 (D0), the HAE-ALI cultures were treated with NU7441 overnight at 5 and 10 μM, respectively, and the media were refreshed the following day. Western blot confirmed the inhibition of DNA-PK_cs_ phosphorylation by the compound at the treated concentration at 16 h post-treatment with NU7441 (Figure 5A). Compared with the vehicle (DMSO) control group, the expression of the transgenes (gLuc and mCherry) in NU7441-treated primary HAE-ALI at 5 and 10 μM was boosted by ∼6- to 9-fold in a dose-dependent manner at D3, D5, D7, and D14 (Figures 5B and 5C). Notably, at working concentrations of 5 or 10 μM, the TEER of the NU7441-treated groups remained >1,500 Ω cm^2^ during the experiment period (14 days), indicating that NU7441 treatment at 5 and 10 μM did not affect the epithelial barrier function (Figure 5D). However, NU7441 at 20 μM reduced the TEER of the treated primary HAE-ALI at 3 days post-treatment (Figure 5D).

Taken together, these results demonstrated that treatment of NU7441 alone significantly increased rAAV transduction of well-differentiated HAE, and NU7441 is able to enhance rAAV transduction synergistically with Dox.

### AZD7648, another DNA-PK_cs_-specific inhibitor, increases rAAV transgene expression in well-differentiated HAE

**Figure 6:**
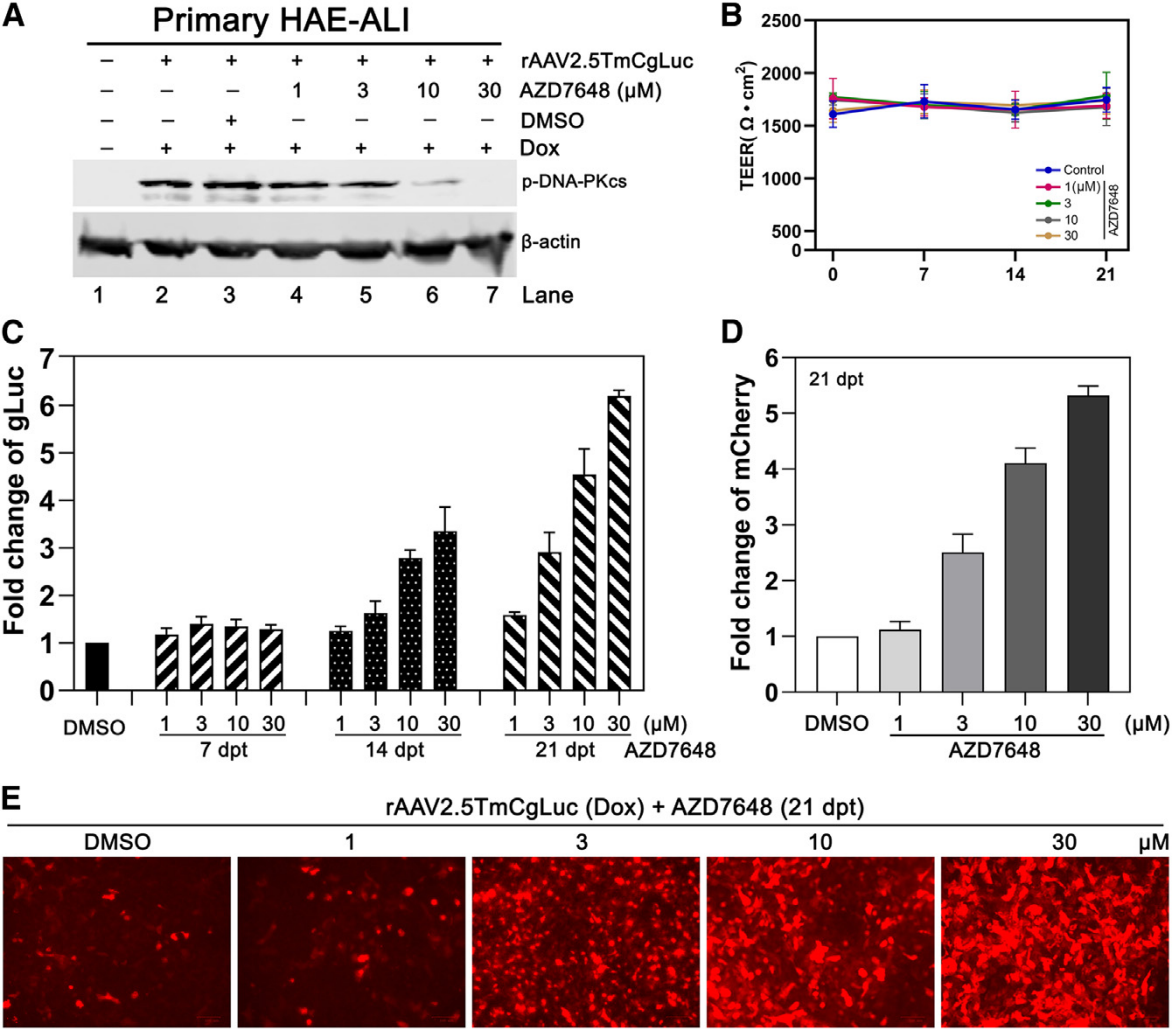
Treatment of AZD7648 increases rAAV transgene expression Primary HAE-ALI cultures, generated from donor B37-22, were treated with AZD7648 at the indicated concentrations for 4 h prior to rAAV2.5TmCgLuc transduction (MOI = 10k) and Dox treatment. After rAAV transduction with Dox overnight, the medium was refreshed without AZD7648. (A) Western blotting. The treated primary HAE-ALI cultures were collected and lysed for western blotting using p-DNA-PK_cs_-specific antibody. DMSO served as a vehicle control. (B) TEER measurement. The TEER values of the treated primary HAE-ALI cultures were measured at 0, 7, 14, and 21 dpt, respectively. Results shown are means and standard deviations (n = 3) from three independent experiments. (C–E) Transgene expression. The expression of gLuc activity was measured at 7, 14, and 21 dpt (C), and mCherry expression was imaged and measured at 21 dpt at the indicated concentration of AZD7648 (D and E).

AZD7648 is a recently developed potent and selective DNA-PK_cs_ inhibitor that is currently under phase 1 clinical trial (ClinicalTrials.gov identifier: NCT03907969).^45^ We further tested its effectiveness on rAAV2.5T transduction in HAE-ALI. We treated primary HAE-ALI (derived from donor B37-22) with AZD7648 at 4 h prior to rAAV2.5TmCgLuc transduction (MOI = 10k), together with 2.5 μM Dox, at 1, 3, 10, and 30 μM, respectively. As expected, phosphorylation of DNA-PK_cs_ induced from the rAAV2.5T transduction was inhibited by the treatment of AZD7648 in a dose-dependent manner, and treatment at 30 μM completely inhibited the activation of DNA-PK_cs_ (Figure 6A). The TEER of treated primary HAE-ALI cultures maintained a value of >1,500 Ω cm^2^ during transduction over the course of 21 days, supporting a low cytotoxicity of AZD7648 at the treated concentration (Figure 6B). Notably, the treatment of AZD7648 resulted in augmentation of gLuc and mCherry expression by ∼6- and ∼5-fold, respectively, compared with vehicle control DMSO group (Figures 6C and 6D).

Collectively, we provide evidence that AZD7648, a potent and selective DNA-PK_cs_ inhibitor undergoing a clinical trial, augmented rAAV transgene expression of HAE, confirming the importance of DNA-PK_cs_ inhibition during rAAV transduction of well-differentiated HAE.

## Discussion

**Figure 7:**
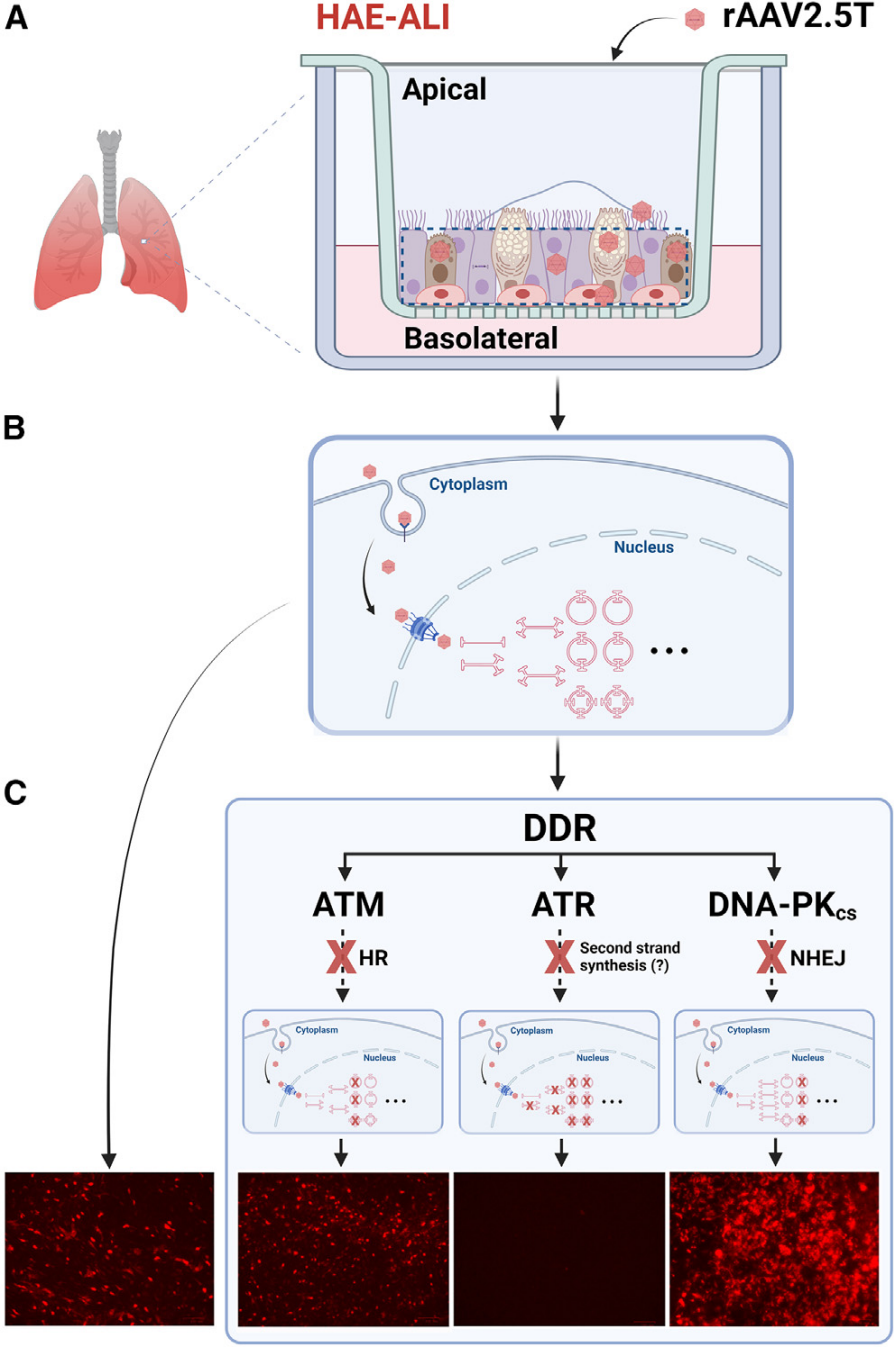
Proposed role of DDR pathways in rAAV transduction of polarized human airway epithelium (A) The schematic diagram of polarized human airway epithelium cultured at an air-liquid interface (HAE-ALI). Human airway epithelial cells isolated from bronchi are cultured at an ALI and differentiated into pseudostratified columnar epithelium composed of multiple epithelial cells. The polarized HAE-ALI enables the study of rAAV2.5T transduction from the apical membrane of the surface airway epithelial cells. (B) The process of apical transduction of rAAV2.5T and genome conversion in polarized epithelial cells. After entry of rAAV2.5T from the apical membrane, the internalized vectors traffic through endosomal processing and enter the nucleus. In the nucleus, the vectors uncoat to release the ssDNA genomes, which are subsequently converted to dsDNA genomes that function in transduction. The dsDNA genomes can be presented in circular or linear forms. The monomer circular episomes form from circularization of the dsDNA viral genomes, and the concatemers form cocatermerization of the dsDNA viral genomes, via homologous recombination (HR) or non-homologous end joining (NHEJ). (C). Proposed role of DDR inhibitions in rAAV2.5T transduction. (1) ATR inhibition or gene silencing inhibits rAAV transduction. As ATR activation is initiated by RPA32 binding of ssDNA breaks and facilitates ssDNA repair, we speculate that the ATR pathway plays an important role in the conversion of the ssDNA genomes to dsDNA. (2) ATM inhibition or ATM silencing has no effects on rAAV2.5T transduction. As the ATM pathway is involved in the DNA repair machinery via HR, we reason that the HR DNA repair pathway is not crucial to the function of episomal or concatermerized rAAV genomes in well-differentiated HAE-ALI. (3) DNA-PK_cs_ inhibition blocks the NHEJ-based repair pathway, which may enhance the formation of episomal or concatemerized dsDNA genomes through an NHEJ-independent manner or simply promotes production of the linear viral dsDNA genomes. X denotes application of inhibitors or gene silencing. The diagram was created using BioRender (biorender.com).

In this study, we provide evidence that rAAV2.5T transduction in well-differentiated HAE induces a DDR signaling with activation of all three PIKKs: ATM, ATR, and DNA-PK_cs_, while there was undetectable host DNA damage. ATR inhibition or *ATR* gene silencing inhibited rAAV transduction, but ATM inhibition or *ATM* silencing had no effects on rAAV2.5T transduction (Figure 7). ATR and ATM are two related kinases that play crucial roles in the cellular DDR pathway, and both play a positive role in wtAAV replication during monoinfection of HEK293T cells.^19^ The distinct effects of the inhibitions of ATR and ATM on rAAV2.5T transduction suggest that the conversion from ssDNA to dsDNA is the rate-limiting step in rAAV transduction.^46^ We reason that the ATR pathway plays a role in this step of rAAV ssDNA genome conversion (Figure 7, ATR). We hypothesize that ATR activation may recruit the necessary DNA repair factors to synthesize the complementary stand of the ssDNA genome as it does in wtAAV replication.^19^ It has been reported that the ITRs of rAAV recruit the MRN complex, which is responsible for the DDR activation.^25^^,^^33^ However, the MRN binding to the ITRs was thought to inhibit rAAV transduction.^33^ The MRN complex is supposed to recruit ATM to the dsDNA breaks and activate ATM.^33^ As ATM inhibition did not increase or decrease rAAV2.5T transduction, we speculate that ATM inhibition would not affect MRN recruitment by the ITRs. Further investigations are warranted to elucidate these steps, including which DNA polymerase is employed in ssDNA conversion, and whether MRN binding to the ITRs inhibits rAAV transduction of differentiated airway epithelia.

rAAV vectors have the ability to persist in the host cell as an episomal circular genome (intramolecular-circularization), dimer, or concatemer (intermolecular-circularization), which allows for long-term expression of therapeutic genes without integrating into the host genome.^47^ NHEJ and HR are the underlying predominant double-strand DNA break (DSB) repair pathway in mammalian cells.^48^ DNA-PK_cs_ is a critical component of the NHEJ repair pathway, which is activated by DSBs and initiates a cascade of events that results in the recruitment of NHEJ-associated proteins to the break site to facilitate the repair process. NHEJ-mediated circularization of rAAV genomes is cell or tissue-type dependent.^49^^,^^50^^,^^51^^,^^52^ Knockout of DNA-PK_cs_ increases linear rAAV genomes in cells but does not increase rAAV transduction.^52^ However, deficiency of Ku80, a key factor of the DNA-PK complex and an essential core component of NHEJ-mediated repair, leads to higher levels of transduced ssDNA rAAV vectors in cell cultures.^32^

Importantly, our study revealed that suppressing the phosphorylation of DNA-PK_cs_ by pharmaceutical inhibitors or targeted silencing significantly increased transgene expression from rAAV2.5T-transduced polarized HAE. DNA-PK_cs_ plays a role in facilitating the formation of circular intermediates during rAAV transduction.^49^^,^^50^^,^^51^ While the dual-AAV vector trans-splicing strategy that relies on the heterodimerization of rAAV genomes was proven efficient to reconstitute an oversized transgene expression in well-differentiated cell types, such as muscle cells, it worked poorly in polarized HAE and mouse lung airways.^53^^,^^54^ The predominant pathway for AAV genome concatemerization is not entirely clear and may depend on various factors. Our previous study showed the importance of ITR sequences in directing intermolecular and intramolecular HR of AAV genomes.^55^ While circularization and heterodimerization of rAAV genomes mediated by the NHEJ mechanism involving DNA-PK may be more efficient than HR-mediated concatemerization, the lack of DNA-PK_cs_ activity in the skeletal muscle of SCID mice did not significantly affect the efficiency of gene expression from the dual-AAV trans-splicing, which relies on the formation of head-to-tail linear and/or circular heterodimer of rAAV genome.^49^ Apparently, further investigations are needed to understand how circularization and heterodimerization of rAAV genomes in polarized airway epithelia are affected by the activation or inhibition of DNA-PK_cs_.

One of the crucial unresolved issues in rAAV vector-based gene therapy pertains to the challenge of achieving sufficient transgene expression while limiting the administered vector dose.^2^ High vector doses would exceed the immunological threshold in humans, potentially leading to toxicity or hazardous immune responses, causing the destruction of the transduced cells.^56^ Pharmacological interventions, such as use of proteasome inhibitors, DNA damage inducer, and epigenetic modifiers, have demonstrated the ability to increase rAAV transduction in dividing or differentiated cells.^41^^,^^57^ The application of high doses of Dox, which is used as a chemotherapy drug by inhibiting topoisomerase II, can induce DNA damage due to its ability to intercalate with DNA in dividing cells.^58^ In our test in the HAE-ALI composed of well-differentiated epithelial cells that are in post-mitotic quiescence, Dox treatment at the working concentration of 2.5 μM induced a DDR as shown by the phosphorylation of RPA32, H2AX, and the three PIKKs, but to a weaker extent than the induction by rAAV transduction. The DDR induced by a combination of rAAV and Dox was not obviously higher than that induced by AAV alone. Dox facilitates rAAV-mediated gene expression both *in vitro* and *in vivo*.^41^^,^^59^ From our results, we reasoned that the Dox-induced DDR negligibly increased the transgene expression, rather it may promote viral nuclear entry through modulation of proteasome function.^41^ Therefore, it is possible that the activation of the DDR pathway by the treatment of Dox and/or rAAV transduction acts as a protective response to activate DNA repair pathways in the well-differentiated epithelial cells. Indeed, under these conditions, we did not detect any obvious damage to the host chromosome DNA of polarized HAE by the comet assay. While the treatment of DNA-PK_cs_ inhibitors enhanced rAAV2.5T transduction in polarized HAE during the infection period synergistically with Dox, it also acts as a “booster” to further increase rAAV transduction a week after Dox treatment, meanwhile, maintaining the epithelial barrier functions. Thus, DDR inhibition by pharmacological interventions enhances rAAV transduction.

Herein, we found that treatment with the DNA-PK_cs_ inhibitors, NU7441 or AZD7648, increased rAAV transduction and boosted transgene expression facilitated by Dox. Importantly, the treatment of NU7441 and AZD7648 alone or in combination with Dox did not significantly increase the internalized rAAV genome (Figure S3). We reason that DNA-PK_cs_ inhibition blocks NHEJ-based repair pathways to enhance the formation of episomal or concatemerized rAAV vectors, which may promote linear viral genome production or NHEJ-independent rAAV genome circulation (Figure 7, DNA-PK_cs_). Although it has been revealed in rAAV-injected muscle,^6^^,^^9^ or cultured cells,^31^ we failed to analyze the forms of rAAV genome in transduced airway epithelia by using southern blotting. This might be due to the length of time in which rAAV vector genomes were evaluated in airway epithelia (21 days) or the limited amount of sample processing. Nevertheless, the function of different forms of rAAV and their relationship with transgene expression is an area that warrants further investigation, for example, by using next-generation sequencing technologies.^60^^,^^61^ Interestingly, pharmacological inhibition of DNA-PK_cs_ can enhance Cas9-mediated genome editing.^62^ NU7441 reduces the frequency of NHEJ while increasing the rate of homology-directed repair following Cas9-mediated DNA cleavage. However, HR is generally inefficient in well-differentiated cell types, such as the airway epithelial cells. rAAV2.5T transduction induced a DDR in HAE-ALI, and the inhibition of DNA-PK_cs_ synergistically works with Dox to augment the transduction. Further investigation into the mechanism of rAAV-induced DDR in polarized HAE might shed the light on the development of CRISPR-based therapeutic genome editing to correct the CFTR mutations in the airways of CF patients.

In summary, this study establishes the benefit of transient NHEJ inhibition with DNA-PK_cs_ inhibitors, NU7441 and AZD7648, for increasing rAAV transduction efficiency in differentiated HAE. This will allow a low vector dose during AAV-based gene delivery to human airways. The combination of DNA-PK_cs_ inhibitors and proteasome inhibitor Dox holds promise to facilitate CF gene therapy using rAAV vectors.

## Materials and methods

### Cell lines

(i) Cell lines: HEK293T cells (CRL-11268) and HeLa cells (CRM-CCL-2) were obtained from ATCC, Manassas, VA. The cells were cultured in Dulbecco’s modified Eagle’s medium (DMEM) (Cytiva Life Science, Marlborough, MA, no. SH30022) with addition of 10% fetal bovine serum (FBS) (MilliporeSigma, St. Louis, MO, no. F0926) at 37°C under 5% CO_2_ atmosphere. CuFi-8 cells are human airway epithelial cells immortalized with *human telomerase reverse transcriptase* and HPV-16 *E6/E7* genes.^44^
(ii) Primary HAE cultures: polarized primary HAE-ALI cultures were generated at the Tissue and Cell Culture Core of the Center for Gene Therapy, University of Iowa.^12^^,^^63^ In brief, human airway (tracheobronchial) epithelial were isolated from the lung of different donors, B13-40, B37-22, and B40-22. Without amplification, the cells were directly cultured on collagen-coated transwell permeable supports (Corning, Corning, NY, no. 3470), and were differentiated at an ALI for 3–4 weeks. The primary HAE-ALI cultures were maintained in (50%/50%) DMEM/F12 medium containing 2% Ultroser G (Sartorius, Goettingen, Germany, no. 15950-017). HAE-ALI cultures were also generated from the immortalized airway epithelial cell lines CuFi-8 (CuFi).^44^ Cells were grown in PneumaCult-Ex Plus Medium (STEMCELL, Vancouver, Canada), and polarized at an ALI with PneumaCult-ALI medium (STEMCELL, no. 05001).

The TEER of the HAE-ALI cultures was measured using an epithelial Volt-Ohm Meter (Millipore). The HAE-ALI cultures that had a TEER value of over 1,500 Ω cm^2^ were chosen for experiments.

### Plasmid constructs

#### pLKO-shRNA constructs

The shRNA-expressing constructs: pLKO-shScram, pLKO-shATM, pLKO-shATR, pLKO-shDNA-PK_cs_ have been described previously.^17^^,^^19^

#### Plasmids for rAAV production

pAAVRep2Cap2.5T, pHelper, rAAV2 *cis* transfer plasmid pAVF5tg83luc-CMVmCherry have been reported previously.^38^^,^^64^ Another *cis* transfer plasmid pAVF5tg83gluc-CMVmCherry was constructed by replacement of fLuc with gLuc in pAVF5tg83luc-CMVmCherry.^64^

### Chemicals and treatments

Hydroxyurea (MilliporeSigma, Burlington, MA, no. 27-07-1) and Dox (Selleckchem, Houston, TX, no. S1208) were prepared according to the manufacturer’s instructions.^41^ Hydrogen peroxide (H_2_O_2_) was purchased from Sigma (St. Louis. MO). PIKKs inhibitors: KU60019 (Tocris Bioscience, Bristol, UK, no. 4176), AZ20 (Selleckchem, no. S7050), NU7441 (Tocris Bioscience, no. 3712), and AZD7648 (Selleckchem, no. S8843) were dissolved in DMSO at 10 mM. The chemicals were applied to cell cultures at the indicated concentrations as we reported previously.^17^^,^^19^^,^^43^

### rAAV production

The production of rAAV was performed following our previously published protocol.^12^ In brief, HEK293T cells seeded in 20- × 150-mm dishes were transfected using PEI MAX (Polysciences, Warrington, PA, no. 24765) at a ratio of 1:3 (plasmid/PEI MAX). A total of 22 μg plasmids (rAAV2 *cis* transfer plasmid, pAAVRep2Cap2.5T, and pHelper [molar ratio of 1:1:1]) were used for each dish. The produced rAAV was then purified and quantified using previously published methods.^12^^,^^64^

### rAAV transduction

rAAV transduction was performed as described previously.^64^ Well-differentiated HAE-ALI cultures were apically transduced by rAAV2.5TmCfLuc (mCherry and firefly luciferase dual-reporter vector) or rAAV2.5TmCgLuc (mCherry and gLuc dual-reporter vector) at the indicated MOI with or without treatment of chemicals. After overnight incubation, the rAAV were removed, and the media were refreshed. The apical chambers were washed by phosphate-buffered saline (PBS) at pH 7. for three times.

### Transgene expression assays

The activity of fLuc was measured with Pierce Firefly Luciferase Glow Assay Kit (Thermo Fisher Scientific, Waltham, MA, no. 16177). gLuc activity was determined using Pierce Gaussia Luciferase glow assay kit (Thermo Fisher Scientific, no. 16161) according to the manufacturer’s instructions. For mCherry expression, HAE-ALI cultures were imaged under the ZOE Fluorescent Cell Imager (Bio-Rad). The fluorescence images were evaluated by using NIH ImageJ software.^65^

### Comet assay

The comet assay was performed using an OxiSelect Comet Assay Kit (Cell Biolabs, San Diego, CA, no. STA-351) according to the manufacturer’s instructions as described previously.^66^ In brief, Dox- and rAAV-treated cells were trypsinized and diluted in PBS. Primary HAE-ALI treated with 100 μM H_2_O_2_ were used as a positive control of DNA damage.^43^ The cells were mixed with 1% low-melting-point agarose and transferred onto slides. After fixing at 4°C, the slides were electrophoresed in alkaline buffer and stained with Vista green dye. The images were captured under a Nikon Eclipse C1 Plus inverted microscope.

### Lentivirus production and transduction

Lentiviruses were produced as previously described.^67^^,^^68^ In brief, HEK293T cells were transfected with the shRNA-expressing pLKO plasmids, together with two packaging plasmids, psPAX2 and pMD2.G, using PEI MAX (Polysciences, Warrington, PA, no. 49553-93-7). The supernatant was collected at 3 days post-transfection and concentrated with a 20% sucrose cushion by ultracentrifugation in a SureSpin 632 rotor (Thermo Scientific) at 19,400 rpm for 3 h. The transduction unit of the produced lentiviruses were titrated as described previously.^69^ Proliferating CuFi-8 cells were transduced at an MOI of ∼5 transduction units/cell. At 3 dpt, the cells were treated with puromycin at a final concentration of 2.5 μg/mL to select the transduced cells.

### Immunofluorescence assay

Immunofluorescence staining was performed as described previously.^70^^,^^71^ In brief, cells of the HAE-ALI cultures were dissociated by incubation with Accutase (Innovative Cell Technologies, San Diego, CA, no. AT-104) for 30 min and washed twice with PBS. The cells were collected and cytospun onto slides at 1,800 rpm for 3 min and fixed with 3.7% PFA at room temperature for 30 min. After washing with PBS three times and permeabilized with 0.5% Triton X-100 for 5 min, the slides were incubated with a primary antibody diluted in PBS with 2% FBS at 37°C for 1 h and subsequently followed with a corresponding secondary antibody. Confocal images were captured under a Leica TCS SP8 STED 3× Super Resolution Microscope. Nuclei were stained with (4′,6-diamidino-2-phenylindole.

### Western blotting

Cells were lysed and separated on sodium dodecyl-sulfate polyacrylamide gel electrophoresis gels along with a protein ladder (GoldBio, St. Louis, MO, no. P008).^18^^,^^72^ The separated proteins were then transferred onto a polyvinylidene difluoride membrane (MilliporeSigma, no. IPVH00010) and blocked with 5% non-fat milk. The membrane was probed with primary and secondary antibodies in order, and the signals were visualized by an Odyssey imaging system (LI-COR Biosciences, Lincoln, NE).

### Antibodies used in the study

#### First antibodies

Rabbit anti-phospho-RPA32 (Thr21) (no. AP1040), rabbit anti-phospho-ATM (Ser1981) (no. AP0008), and rabbit anti-phospho-DNA-PK_cs_ (Ser2056) (no. AP0621) were purchased from Abclonal (Woburn, MA). Rabbit anti-phospho-ATR (Thr1989) (no. GTX128145) were purchased from GeneTex (Irvine, CA). Mouse anti-ꝩH2AX (Ser139) (no. 05-636) and mouse anti-β-actin (no. A5441) were purchased from MilliporeSigma.

#### Secondary antibodies

DyLight 800-conjugated anti-rabbit IgG (no. 5151S) and DyLight 800-conjugated anti-mouse IgG (no. 5257S) were purchased from Cell Signaling (Danvers, MA); Alexa Fluor 488-conjugated anti-rabbit IgG (no. 111-545-003) and Alexa Fluor 488-conjugated anti-mouse IgG (no. 115-545-003) were purchased from Jackson ImmunoResearch (West Grove, PA).

### Statistical analysis

Data shown are means and standard deviations and are representative of three independent experiments. Statistical analyses were performed using GraphPad Prism 9 (GraphPad Software, San Diego, CA). p values of statistical significance were analyzed using Student’s t test. ∗∗∗∗p < 0.0001, ∗∗∗p < 0.001, ∗∗p < 0.01, and ∗p < 0.05 were regarded as statistically significant and n.s. represents no statistical significance.

## Data and code availability

The data that support the findings of this study are presented in the paper and/or the supplemental materials.

## Supplemental information

Document S1. Figures S1–S3 and supplemental materials and methods

Document S2. Article plus supplemental information
