# Supplementary material for: Inhibition of DNA-dependent protein kinase catalytic subunit boosts rAAV transduction of polarized human airway epithelium
Source: Mol Ther Methods Clin Dev. 2023 Sep 21;31:101115. doi: 10.1016/j.omtm.2023.101115 (PMC10568418; doi:10.1016/j.omtm.2023.101115)
Supplement: Document S1. Figures S1–S3 and supplemental materials and methods [file mmc1.pdf]

OMTM, Volume *31*

Supplemental information

Inhibition of DNA-dependent protein kinase catalytic subunit boosts rAAV transduction of polarized human airway epithelium

Kang Ning, Xiujuan Zhang, Zehua Feng, Siyuan Hao, Cagla Aksu Kuz, Fang Cheng, Soo Yuen Park, Shane McFarlin, John F. Engelhardt, Ziying Yan, and Jianming Qiu

Supplemental Materials

Materials and Methods:

rAAV internalization assay.

Well-differentiated primary HAE-ALIs cultures were transduced with rAAV2.5TmCgLuc at an MOI of 10 K. DOX (2.5 µM), NU7441 (2 µM), and AZD7648 (10 µM) were added into the media and DMSO served as vehicle control. After overnight incubation, the cells were washed with PBS three times. The attached viruses were removed by treatment with Accutase (#AT-104, Innovative Cell Technologies, San Diego, CA) at 37°C for 15 min and washed with PBS 3 times. The total DNA was extracted by using a ZYMO Quick-DNA/RNA Pathogen Miniprep kit (#R1043, ZYMO Research, Irvine, CA). The internalized rAAV genomes were quantified by qPCR with a mCherry specific probe (5'6-FAM [Fluorescein]/TTC AAG TGG/ZEN/ GAG CGC GTG ATG AA/3'IABkFQ) and primers (Forward: 5'-GAC TAC TTG AAG CTG TCC TTC C-3'; Reverse: 5'-CGC AGC TTC ACC TTG TAG AT-3').

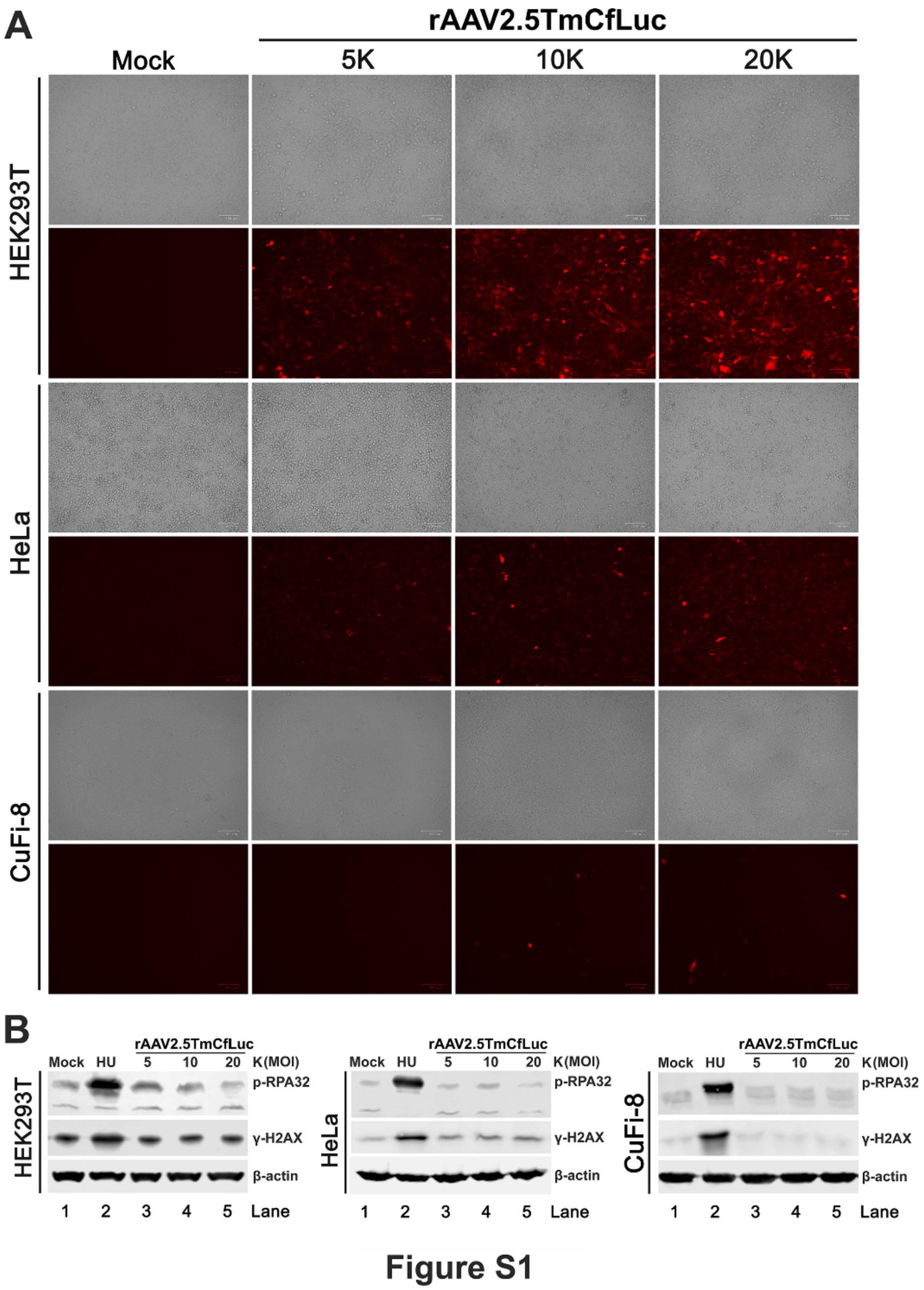

Figure S1. rAAV transduction induces negligible DDR signals in HEK293T, HeLa and dividing CuFi-8 cells.

HEK293T, HeLa and CuFi-8 cells were transduced by rAAV2.5TmCfLuc at an MOI of 5, 10, and 20 K, respectively. **(A) mCherry expression.** The indicated cultures were imaged for mCherry expression at 3 dpt under the ZOE Fluorescent Cell Imager (BioRad). **(B) Western blotting.** At 3 dpt, the cells were collected and lysed for the detection of phosphorylated RPA32 and H2AX with specific antibodies. β-actin served as a loading control.

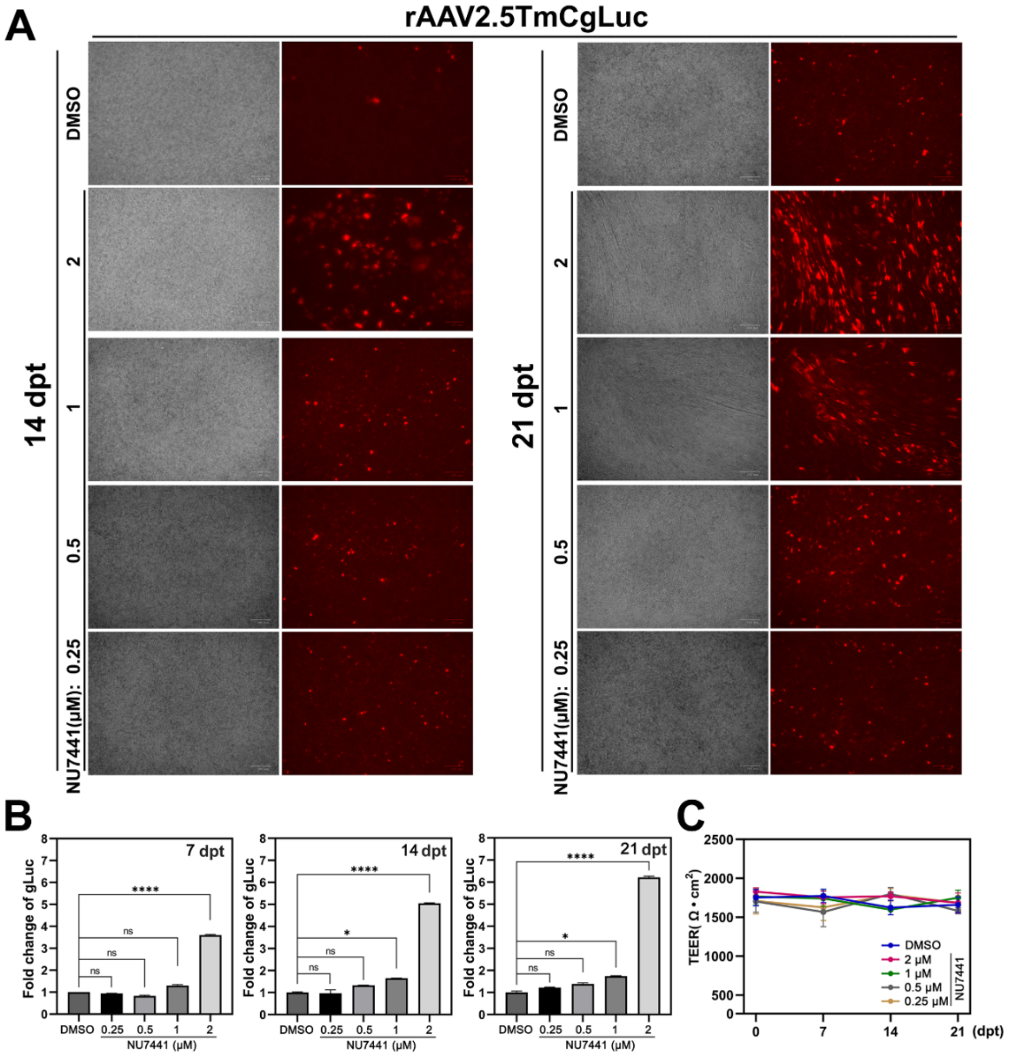

Figure S2

Figure S2. Treatment of NU7441 increases rAAV transduction of primary HAE-ALI cultures and is donor independent.

Primary HAE-ALI cultures (B40-22) were treated with NU7441 for 4 h at indicated concentrations and transduced by rAAV2.5TmCgLuc at an MOI of 10K, together with Dox at 2.5 µM. DMSO was used as a vehicle control. **(A&B) Transgene expression.** At 14 and 21 dpt, expression of mCherry was imaged, respectively (A). The fold change of gLuc activity in NU7441 treated groups was normalized to the DMSO control group at 7, 14, and 21 dpt (B). **(C) Measurement of TEER.** At 0, 7, 14, and 21 dpt, the TEER of DMSO and NU7441 treated primary HAE-ALI cultures were measured. Results shown are means and standard deviations (n = 3). P values were calculated by using Student's t test (*, P<0.05; ****, P<0.0001; n.s., no statistically significant difference).

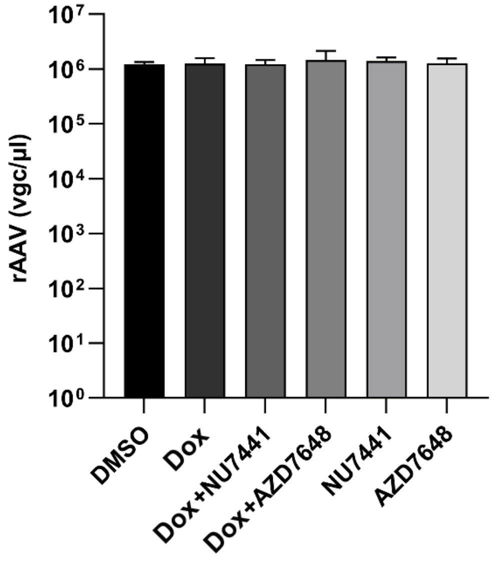

Figure S3

Figure S3. Treatment with DNA-PKcs inhibitors alone or combined with Dox (doxorubicin) does not affect rAAV vector internalization.

Well-differentiated primary HAE-ALI cultures were transduced with rAAV2.5TmCgLuc at an MOI of 10 K. Dox (2.5 µM), NU7441 (2 µM), and AZD7648 (10 µM) were added into the media and DMSO served as vehicle control. After overnight incubation, the cells were washed with PBS three times, and the attached viruses were removed by Accutase treatment. The internalized rAAV genomes were quantified by qPCR with a mCherry specific probe and a pair of primers. Results shown are means and standard deviations (n = 3) from three independent experiments.
